# An Angular Acceleration Based Looming Detector for Moving UAVs

**DOI:** 10.3390/biomimetics9010022

**Published:** 2024-01-02

**Authors:** Jiannan Zhao, Quansheng Xie, Feng Shuang, Shigang Yue

**Affiliations:** 1 Guangxi Key Laboratory of Intelligent Control and Maintenance of Power Equipment, School of Electrical Engineering, Guangxi University, Nanning 530004, China; jzhao@gxu.edu.cn (J.Z.); chasen1999xqs@outlook.com (Q.X.); 2 School of Computing and Mathematical Sciences, University of Leicester, Leicester LE1 7RH, UK; sy237@leicester.ac.uk

**Keywords:** Bio-inspired Neural Networks, collision detection, dynamic vision, LGMD, UAV

## Abstract

Visual perception equips unmanned aerial vehicles (UAVs) with increasingly comprehensive and instant environmental perception, rendering it a crucial technology in intelligent UAV obstacle avoidance. However, the rapid movements of UAVs cause significant changes in the field of view, affecting the algorithms’ ability to extract the visual features of collisions accurately. As a result, algorithms suffer from a high rate of false alarms and a delay in warning time. During the study of visual field angle curves of different orders, it was found that the peak times of the curves of higher-order information on the angular size of looming objects are linearly related to the time to collision (TTC) and occur before collisions. This discovery implies that encoding higher-order information on the angular size could resolve the issue of response lag. Furthermore, the fact that the image of a looming object adjusts to meet several looming visual cues compared to the background interference implies that integrating various field-of-view characteristics will likely enhance the model’s resistance to motion interference. Therefore, this paper presents a concise A-LGMD model for detecting looming objects. The model is based on image angular acceleration and addresses problems related to imprecise feature extraction and insufficient time series modeling to enhance the model’s ability to rapidly and precisely detect looming objects during the rapid self-motion of UAVs. The model draws inspiration from the lobula giant movement detector (LGMD), which shows high sensitivity to acceleration information. In the proposed model, higher-order information on the angular size is abstracted by the network and fused with multiple visual field angle characteristics to promote the selective response to looming objects. Experiments carried out on synthetic and real-world datasets reveal that the model can efficiently detect the angular acceleration of an image, filter out insignificant background motion, and provide early warnings. These findings indicate that the model could have significant potential in embedded collision detection systems of micro or small UAVs.

## 1. Introduction

Real-time robust collision detection provides critical environment sensing and decision support for animal and robot behaviors and tasks. However, the current mainstream collision detection algorithms based on distance and image trends have proven inadequate for meeting UAVs’ low-power sensitive collision detection requirements. This limitation significantly impairs the performance and applicability of UAVs in tasks such as collision avoidance and navigation. For instance, the technique of utilizing laser [1], ultrasonic [2], and other sensors to measure the relative distance trend between the UAV and the obstacle to caution against collisions is inadequate in presenting adequate information about the obstacle’s position, shape, and boundary. This shortfall will impede the UAVs’s accuracy and flexibility in avoidance maneuvers. Methods that use visual sensing capture rich information about the natural world (including the obstacle shape, position, and boundary information) and combine it with deep learning [3] to analyze the relationship between the position of the calibrated obstacle in the pre-collision image and the TTC, as well as the relative distance, to warn of collisions. However, this method does not clarify the relationship between the looming result and the image. Its interpretability and environmental adaptability are poor, and it performs poorly in the face of new background environments and obstacles. On the other hand, if we can quickly extract the critical looming cues from image changes during looming to predict collisions like insects [4], combined with the rich object information given to us by vision, We then have the potential to create a low-power, real-time, robust looming detection algorithm.

A substantial amount of biobehavioral experiments have demonstrated that looming-sensitive neurons in insects [4,5,6], which are responsible for alerting them of potential collisions, can detect and react to various looming visual stimuli. It is thought that the neural computations that encode these diverse looming visual characteristics and generate avoidance commands are crucial for developing efficient looming perception in insects. LGMD neurons are particularly useful in constructing looming-sensing systems due to their maximum response to looming [4,7] and continuously increasing visual stimuli [8], their transient response to retreating objects [9], and their resistance to interference from random motion backgrounds [10]. However, the implementation of these bionic algorithms in fast-moving UAVs is impeded by changes in images caused by the UAV’s motion. This affects the model’s ability to accurately extract collision visual features, resulting in a high false alarm rate and warning time lag.

**Figure 1:**
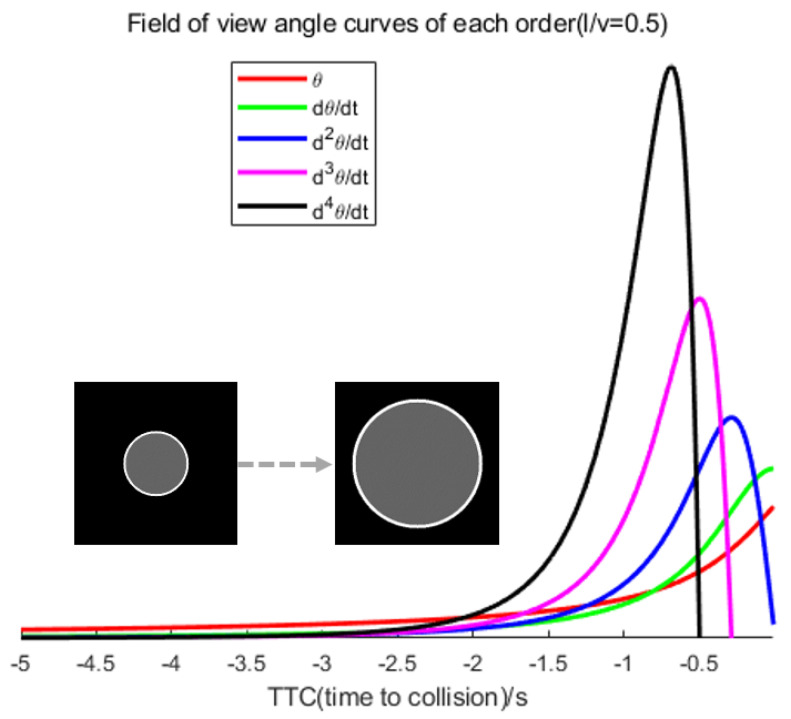
Visual field angle curves of different orders based on Equations (A1)–(A3) (only plotting the segments where the curves are greater than 0). Here, length/velocity(l/v)=0.5 . The dθ⁄dt and θ curves monotonically increase until a collision occurs. For second-order and higher visual field angle curves, there will be a peak, exhibiting a linear relationship with l/v .

Therefore, we must identify a prominent visual cue that is not affected by the angular velocity of the image to counteract the interference caused by the observer’s motion and advance the warning time. Before the collision, it was observed that peaks emerged in the higher-order field of view curves (second order and above) while plotting the curves for each order. The time of the peaks appeared to be linearly related to l/v . Additionally, the higher the field of view curves, the earlier the peaks occurred (for example, the second-order derivative of Equation (A5), where $t_{spike}=0.577l/v$ , and the third-order derivative, where $t_{spike}=l/v$ ). This indicates that encoding higher-order field-of-view data from the image and issuing an early warning when the peaks in the higher-order field-of-view curves manifest would theoretically grant the controller ample time to evade. In Figure 1, objective observation is that, unlike other higher-order field-of-view curves, the second-order field-of-view curve (field-of-view angular acceleration) consistently has a numerical value greater than zero. This implies an uninterrupted increase in the field-of-view angular velocity and angle. On the other hand, organisms can sense displacement (visual field angle) through photoreceptors and then indirectly sense changes in velocity (visual field angular velocity) based on the passage of time, and there are also peaks in the true potential curves in locust LGMD neurons. It is probable that biological neurons sensitive to looming encode angular acceleration through the angle and velocity and utilize peaks in the angular acceleration curve as collision warnings.

Inspired by this, we propose the A-LGMD model, which can extract acceleration information and combine the angular size and angular velocity information in real-time to accomplish the task of collision detection in a motion scene. Specifically, we propose a two-channel distributed synaptic structure to filter two sets of image angular velocity information with a time delay. We then activate and aggregate the two sets of angular velocity information in the local spatiotemporal domain to obtain the image angular acceleration information. Finally, we fuse multiple looming visual cues and adopt the “peak” triggering method to warn the image of a significant collision. The model serves to warn of substantial looming objects within the image. A systematic evaluation of the model is conducted, encompassing the retrieval of angular acceleration information from the picture, as well as a comparison with other bionic looming detection models in simulated and real UAV flight videos. The study’s main contributions comprise several facets.

1. We deduce that there exist peaks in the curves of the high-order (≥2) differentials of the angular size of a looming object. In particular, the peaks of these curves occur earlier as the differential order increases. This suggests that it is worth increasing the alarming TTC of a looming detection model by introducing higher-order information on the angular size.
2. Based on the D-LGMD model, which is an angular velocity-focused algorithm, we introduce the angular acceleration cues, intending to increase sensibility and acquire an earlier alarm time before a collision.
3. We have conducted a systematic analysis and comparison of the performance of the proposed A-LGMD model with various other bionic looming detection models in different scenarios. The experimental results demonstrate that the proposed model has a distinct tendency toward looming objects in the observer’s motion and is capable of fulfilling the collision detection task of UAVs.

This paper is organized into several sections. Section 2 outlines the related research. Section 3 provides a comprehensive explanation of the A-LGMD model. Section 4 presents the results of experiments that demonstrate that the algorithm proposed in this paper surpasses other bionic looming algorithms in identifying looming objects amidst camera self-motion. Lastly, our conclusions are drawn in Section 5.

## 2. Related Work

In this section, we review the bionic looming detection algorithms based on motion vision, followed by an introduction to the properties of LGMD neurons and their bionic looming detection models, which are relatively well-researched, and finally a summary of the related research on looming visual cues.

### 2.1. Looming Visual Cues

Looming detection is a scientifically inspired principle for identifying potential collision risks by observing the visual processes of approaching objects [11]. Animals have been observed to be able to predictively perceive collisions through different sensitive neurons. For instance, in pigeons, certain neurons, namely τ , η , and ρ [12], respond to the time to collision (TTC) at distance [13]. In locusts, LGMD neurons are sensitive to image angles and angular velocities, while in *Drosophila*, lobula plate/lobula columnar type II (LPLC2) [4] is sensitive to radially symmetric motion.

In investigating looming processes, numerous scholars have examined the correlation between the looming bias of responsive neurons and changes in obstacle images whilst looming. For instance, Rind and Zhao contend that the angular size of obstacles on the retina and the angular velocity consistently increase during looming. Rind utilized the LGMD model [14] to extract the fast-moving edges of the looming objects and set the angular size threshold for collision identification. Zhao, on the other hand, extracted the image angular velocity using the D-LGMD model [15] and established the angular velocity threshold for collision identification. Both approaches were employed for collision detection. Baohuazhou and John Stowers conducted a study on the *Drosophila* visual system and proposed that during the looming process, the motion of an obstacle on the observer’s retina creates locally symmetric information. Based on the *Drosophila*’s opponency ultra-selectivity [16], Baohuazhou developed a shallow neural network model [17] that is constrained anatomically to recognize the looming signal. Similarly, Zhao asserted that *Drosophila* determine collisions based on the radial symmetry of objects and developed the opponency model [18]. John Stowers utilized the dispersion of optical flow vectors at the focus of expansion to determine the approximate position of looming objects, based on the characteristic that *Drosophila* strongly avoid the focus of the optical flow expansion when moving toward an object [19]. This allowed for achieving the obstacle avoidance function of UAVs.

These bionic algorithms accurately depict the pre-collision process by analyzing the characteristics of the looming object in the image, which is superior to distance-based collision detection algorithms.

### 2.2. Bionic Looming Detection Algorithms

Researchers have harnessed the biological mechanism of looming neurons to develop multiple bionic proximity detection algorithms, which utilize the image difference principle. These algorithms aim to effectively detect looming by encoding visual cues in the differences between consecutive frames and establishing early warnings based on different visual cues.

Rind put forward the concept of excitatory-inhibitory critical competition, drawing on the preference of locust LGMD neurons for visual stimuli that are looming and continuously increasing. Rind developed a computational model of LGMD1 which aligns with the locust LGMD neuron characteristics, such as transient excitation due to changes in light [14], lateral inhibition [4], and global inhibition [20]. LGMD1 extracts the velocity and number of object motion edges from differential images to indicate the degree of approaching and enable selectivity for approaching objects. Scholars have made various improvements and refinements to the LGMD model due to biologists’ deeper exploration of LGMD properties and the challenges encountered by the LGMD model in robotic applications. These include the following. To enhance the resilience of the LGMD model to ambient light interference, Fu and Yue [21] constructed the LGMD2 model based on its characteristic persecution selectivity for changes between light and dark. This was achieved by including on and off channels in the photoreceptor layer of the LGMD2 model, which separates the image’s brightness changes into on and off channels (on meaning from dark to light and off meaning from light to dark). Lei [22] proposed a new model for LGMD1 that relies on neural competition between the on and off pathways to discern fast translational motion objects. The model is not receptive to paired on-off responses triggered by translational motion, hence improving collision selectivity. To adapt the LGMD1 model for UAV flight scenarios, Zhao proposed a distributed presynaptic connectivity structure [15] grounded in LGMD1. The structure effectively removes background motion noise by precisely extracting the image’s angular velocities.

Inspired by the ultra-selective, looming-sensitive neuron LPLC2 in the *Drosophila* visual system, Zhao [18] proposed a definition for radially symmetric motion, utilizing the lateral inhibition mechanism to extract the motion velocity and radial symmetry in four directions. This generates a map of the self-attention mechanism. The opponency-based looming detector (OppLoD) model, as proposed by Zhao, integrates the D-LGMD output with the map to extract radially symmetrical motion information that contains faster motion speeds.

### 2.3. LGMD Neuron Properties and Model Applications

The LGMD is a visual neuron specializing in collision detection in the locust visual system. Its characteristic looming selectivity derives from its dendritic fan [23]. This area responds maximally to visual stimuli that are looming [4] and consistently increasing in magnitude [8]. It responds only briefly to retreating objects [9] and is also resistant to interference from random motion backgrounds [10,24], and it is closely linked to downstream motor neurons and the downstream contralateral motion detector (DCMD) [4] to initiate an escape response [25].

In robotics experiments, Yue and Rind included group excitation and attenuation processing layers in the LGMD1 model [26] to increase the robustness and adaptability to dynamic backgrounds [11]. For UAV applications, Zhao et al. suggested an LGMD1 model built on distributed presynaptic connectivity (D-LGMD1) [15], which employs spatiotemporal filters in lateral inhibition and is useful in situations involving high-speed camera movement. Lei et al. exhibited an improved LGMD1 model featuring on and off dual paths [22]. By assessing the outcome of the fusion of both routes, the model proficiently subdued the reaction to translational movement.

## 3. Method

In this section, we present an acceleration looming motion detector (A-LGMD) that is based on the D-LGMD model. To extract acceleration information from the velocity space, the model introduces a dual-channel distributed synaptic front-end connection for encoding two sets of angular velocity information. Afterward, the model undergoes spatiotemporal delay activation aggregation for the angular velocity information to obtain angular acceleration information from the image. Considering the continuity of neural processes, the model has been transformed into a continuous integration form. Additionally, the threshold processing has been optimized and adjusted to improve its performance and stability while maintaining the model’s proximity detection selectivity in the presence of complex background motion, as shown in Table 1.

### 3.1. Mechanism and Schematic

From the previous analyses, it is evident that the image of the ideal looming object displays a sharp, nonlinear expansion prior to a collision. The angular velocity and acceleration of the image both exhibit nonlinear increases, with the moment of the sharp peak of the angular acceleration curve being linearly linked to l/v , as displayed in Figure 1. Although the angular acceleration and angular velocity present information differently, the acceleration information is considered to be of a higher order than the velocity information. To perceive the angular acceleration, algorithms that encode the angular velocity can be used as inspiration. We have developed a method of excitation inspired by the D-LGMD model which competes with excitation and inhibition to filter and retain different velocity information [20]. Subsequently, delayed activation and aggregation of various velocity information are executed to generate data on the image’s angular acceleration. Furthermore, the field of view, image angular velocity, and image angular acceleration are nonlinearly coupled and taken as inputs to the Izhikevich neuron model [27]. A “peak” triggering mechanism is utilized to indicate the existence of noteworthy looming objects in the frame.

**Figure 2:**
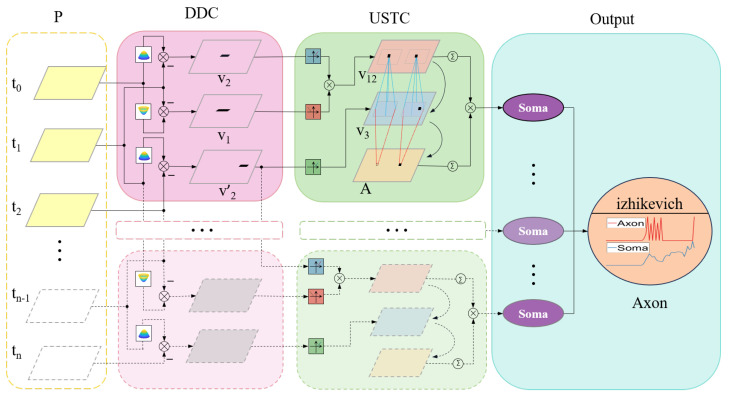
Model schematic. P: photo-receptor; DDC: distribution dual channel; USTC: ultra-spatiotemporal connection. The Output comprises two layers: the Soma layer and the Axon layer. The Soma layer integrates the looming information from the USTC layer, while the Izhikevich impulse neurons in the Axon layer generate the impulse information. This model extracts potential danger from image sequences and produces a sequence of pulses that warn of obstacles.

Through the above design, the A-LGMD model presents several advantages, such as improved accuracy in recognizing looming objects, faster response to looming objects, and better resistance to interference from background motion. The structure of the A-LGMD model is explained in detail in Figure 2. This model consists of three image-processing steps.

- Dual-channel extraction of angular velocity from images;
- The activation of angular velocity information is delayed, allowing for the aggregation of angular acceleration information;
- Multiple cues for looming stimuli are fused, and warning signals are triggered by peaks.

In the P layer of photoreceptors, image motion information is extracted. Within the DDC layer, motion information undergoes excitation and inhibition competition based on the rind key competition concept, resulting in two distinct sets of image angular velocity information. In the USTC layer, delay activation and aggregation are utilized on two distinct velocity information sets to acquire insights into the angular acceleration of the image. Various looming clues are combined and outputted in the Soma layer. Lastly, the axon layer adopts a “peak” triggering mechanism to signify objects of importance looming in the scene.

### 3.2. Photoreceptor Layer

The initial layer of the model comprises the photoreceptor layer (P layer). Track alterations are in the overall luminosity at every pixel point:

(1) $$P\left(x,y,t\right)=\left|L(x,y,t)-\int L(x,y,s)\delta (t-s-1)ds\right|$$

(2) $$\delta \left(t\right)=\left\{\begin{array}{cc} 1 & t=0\\ 0 & otherwise \end{array}\right.$$

where δ is a unit impulse function, L(x,y,t) denotes the brightness alteration at a certain time, and P(x,y,t) represents the overall brightness alteration of the pixel (x,y) over a time *t*. Layer P embodies variations in the brightness of the image motion regardless of direction.

### 3.3. Distribution Dual-Channel Layer

**Figure 3:**
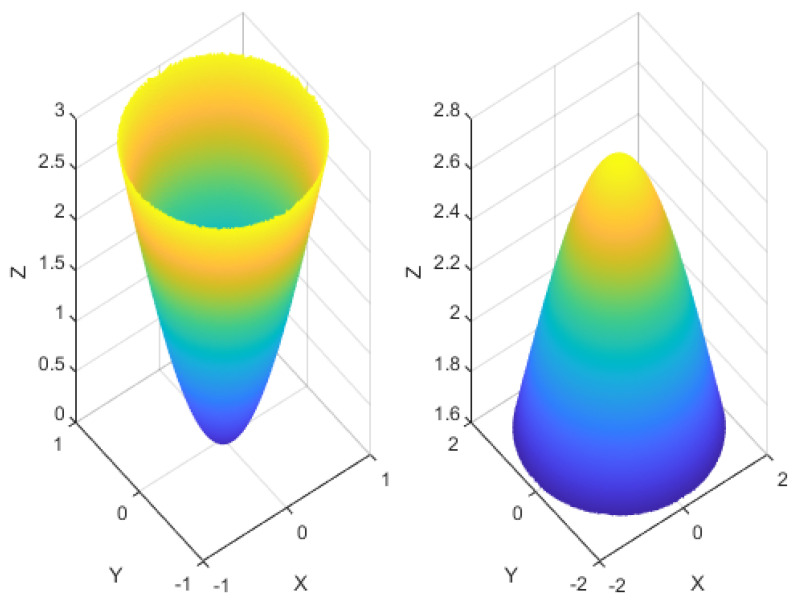
Examples of the low-speed and high-speed inhibitory kernels $K_{I_{1}}$ and $K_{I_{2}}$ , respectively. The left image shows the kernel grid of $K_{I_{1}}$ , while the right image displays the kernel grid of $K_{I_{2}}$ , where $\sigma_{1}=r1=1$ and $\sigma_{2}=r2=2$ .

In the DDC layer, two pathways are present: one with distributed excitatory properties and the other with distributed inhibitory properties, which compete over time to extract two distinct angular velocities and transmit them to the subsequent USTC layer. By employing a mechanism of temporal competition between excitatory and inhibitory signals, two distinct angular velocity representations, $V_{p1}$ and $V_{p2}$ , can be obtained for each pixel point.

(3) $$V_{p_{1}}\left(x,y,t\right)=\int E\left(x,y,s\right)\delta \left(t-s-1\right)ds-\int I_{1}\left(x,y,s\right)\delta \left(t-s-2\right)ds$$

(4) $$V_{p_{2}}\left(x,y,t\right)=\int E\left(x,y,s\right)\delta \left(t-s\right)ds-\int I_{2}\left(x,y,s\right)\delta \left(t-s-1\right)ds$$

where E(x,y,t) and I(x,y,t) represent the signals of excitation and inhibition for each pixel extracted from the edge information of the image motion, respectively, and

(5) $$E\left(x,y,t\right)=\int P\left(x,y,t\right)K_{E}\left(x-u,y-v\right)dudv$$

(6) $$I_{i}\left(x,y,t\right)=\int P\left(x,y,t\right)K_{I_{i}}\left(x-u,y-v,t-s\right)dudvds$$

where $K_{E}$ and $K_{I}$ are the distribution functions of excitation and inhibition, respectively, and $K_{I}$ is distributed over time and space. Given the global and local inhibition properties of LGMD neurons, it is physiologically inferred that the presynaptic neighbors transmit inhibition. We have theorized that the dendritic inhibition strength of LGMD neurons possesses a shape characteristic that gradually decreases from the tip of the branch crystal to the root along its diameter [28,29]. This characteristic could account for the local inhibition at the distal end [9]. Consequently, we have chosen to illustrate the spatial distribution of $K_{I}$ by using an inverted Gaussian kernel with equivalent traits. The inverted Gaussian kernel $K_{I_{1}}$ obstructs faraway motion information, therefore favoring low speeds. The Gaussian kernel $K_{I_{2}}$ obstructs nearby motion information, thus opting for high speeds. The low-speed and high-speed inhibitory as shown in Figure 3.

(7) $$\left\{\begin{array}{c} K_{E}=G_{\sigma_{E}}\left(x,y\right)\\ K_{I_{i}}=G_{\sigma_{I}}\left(x,y\right)\delta \left(t-\tau_{i}\left(x,y\right)\right) \end{array}\right.$$

where $\sigma_{e}$ and $\sigma_{i}$ represent the standard deviation between the excitation and inhibition, respectively, while $\tau_{i}\left(x,y\right)$ is the time-mapped function of the inhibition pathway. The delay is determined by *r* and increases as the transmission distance increases as shown in the subsequent equation:

(8) $$\left\{\begin{array}{c} G_{\delta }=\frac{1}{2\pi \sigma^{2}}e^{-\frac{x^{2}+y^{2}}{\sigma^{2}}}\\ \tau_{i}\left(x,y,r\right):x^{2}+y^{2}<r_{i}^{2} \end{array}\right.$$

**Figure 4:**
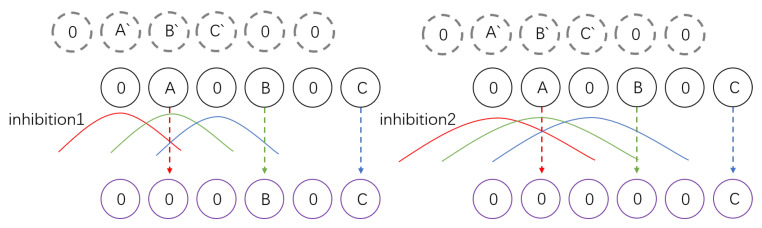
$A^{\prime }$ position from the previous time step is denoted. Pixels A, B, and C move at speeds of 1, 2, and 3 pixels/ms, respectively. The inhibition1 and inhibition2 radii indicate the inhibition ranges of inhibition pathways 1 and 2. The inhibition values generated by $A^{\prime }$ , $B^{\prime }$ , and $C^{\prime }$ are represented by red, green, and blue colors, respectively. When the movement of the pixel generates a level of stimulation beyond its inhibition threshold, the DDC layer will receive speed data above the threshold (resulting in a purple output).

In the tao function, *r* denotes the spatial range of inhibition, which sets the lowest speed of the inhibition channel during the competition with excitation. By adjusting the width of tao for various inhibitory channels, it is viable to screen out data with velocities exceeding *r* as depicted in Figure 4.

### 3.4. Ultra-Spatiotemporal Connection Layer

**Figure 5:**
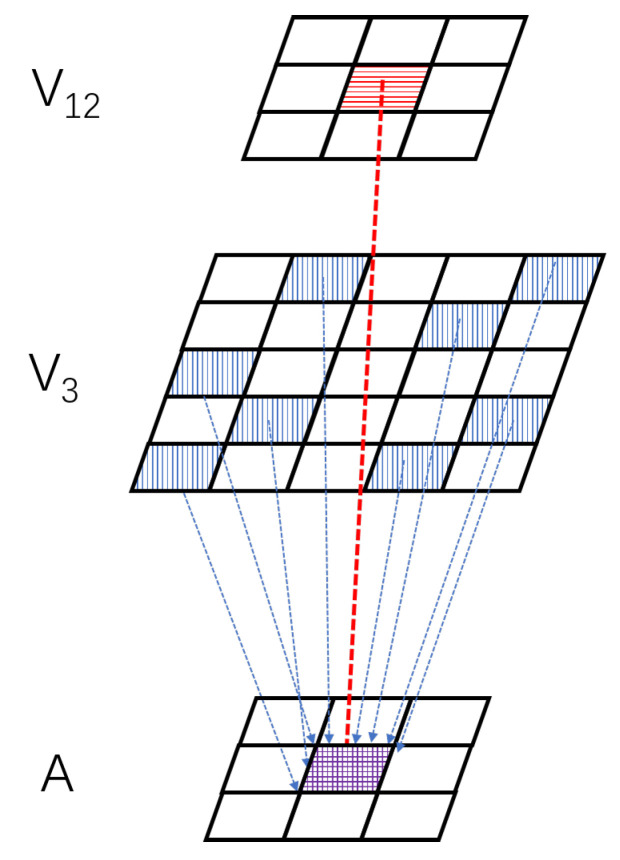
Schematic for collecting acceleration data within the USTC layer. The USTC layer generates acceleration information by aggregating data on high-speed moving pixels (blue pixels) near the pre-accelerated pixels (red pixels).

To extract information on the angular acceleration, the USTC layer receives angular velocity data from the DDC layer at various points in time. This velocity data are then delayed and compiled to form “acceleration information” in the local space, as shown in Figure 5. In the USTC layer, the coordinates of the pixel with an angular velocity between v1 and v2 are determined by processing the two velocities at the same time using two opposite polarity activations, as detailed in Equation (12). Next, the possible acceleration location of the pixel is aggregated within the DDC layer’s aggregation region and outputted to the A layer as the weight of the pixel’s acceleration impact, as shown in Equation (9). Equation (9) can also be interpreted as the pixel points where the image angular velocity jumps within a specified velocity interval ( v1 – v2 ) over a given time interval. With this design, it is possible to capture the information on accelerated motion in the image effectively. See the following equation for more information:

(9) $$A\left(x,y,t\right)=\left\{\begin{array}{cc} \underset{\tau_{3}\left(x,y,r_{3}\right)}{\;\int \int \;}V_{3}\left(x,y\right)dxdy & V_{12}\left(x,y,t\right)>0\\ 0 & otherwise \end{array}\right.$$

Since the radius of $r_{3}$ ( $r_{3}=2r_{2}-r_{1}+1$ ) is larger than $r_{2}$ , Equation (9) aggregates the pixel points ( $V_{12}$ ) with velocities less than r2 at the previous moment within the radius of r3 around the noteworthy pixel points ( V3 ) with angular velocities greater than r2 . Here, V3 indicates the noteworthy pixel points, and V12 represents pixel points with angular velocities between v1 and v2 , as shown in Equations (10) and (11):

(10) $$V_{3}\left(x,y,t\right)=\max\left(0,V_{p_{2}}\left(x,y,t\right)-k_{3}\right)$$

(11) $$V_{12}\left(x,y,t\right)=V_{1}\left(x,y,t\right)\times V_{2}\left(x,y,t\right)$$

where V1 and V2 represent the angular velocity data for the pixel with varying velocity limits at the preceding instant. V1 is used to exclude minor background movements, while V2 signals the presence of a significant movement that merits attention:

(12) $$\left\{\begin{array}{c} V_{1}\left(x,y,t\right)=max\left(0,V_{p1}\left(x,y,t\right)-k_{1}\right)\\ V_{2}\left(x,y,t\right)=max\left(0,-V_{p_{2}}\left(x,y,t-1\right)-k_{2}\right) \end{array}\right.$$

### 3.5. Soma Layer

To mitigate the impact of the robot’s motion on the encoded angular velocity, we employed the image angular acceleration (higher-order information) to offset the image angular velocity (lower-order information). This is akin to applying triple correlation to eliminate variability in the second-order components and enhance the motion estimation accuracy [30]. From Equation (A3), it is clear that the ttc estimation has a positive correlation with the angular velocity and an inverse proportion to the field of view angle. Therefore, including both angular velocity and field of view angle information is essential for warning about collisions earlier at TTC moments in the proximity detection model. On the other hand, estimating the field of view angle significantly relies on the robot’s motion. Consequently, we only combined the angular velocity (which includes motion edge information (i.e., the field of view angle)) and angular acceleration into the output of the Soma layer as illustrated in Equation (13):

(13) $$Soma\left(t\right)=\iint A\left(x,y\right)dxdy\iint V_{3}\left(x,y\right)dxdy$$

This leads to a Soma output that is more attuned to objects with a wider field of view angle and faster motion while being less responsive to extraneous objects and background noise. Ultimately, the global acceleration motion information and velocity information in the USTC layer are nonlinearly merged to form the output of the Axon layer.

### 3.6. Axon Layer

**Figure 6:**
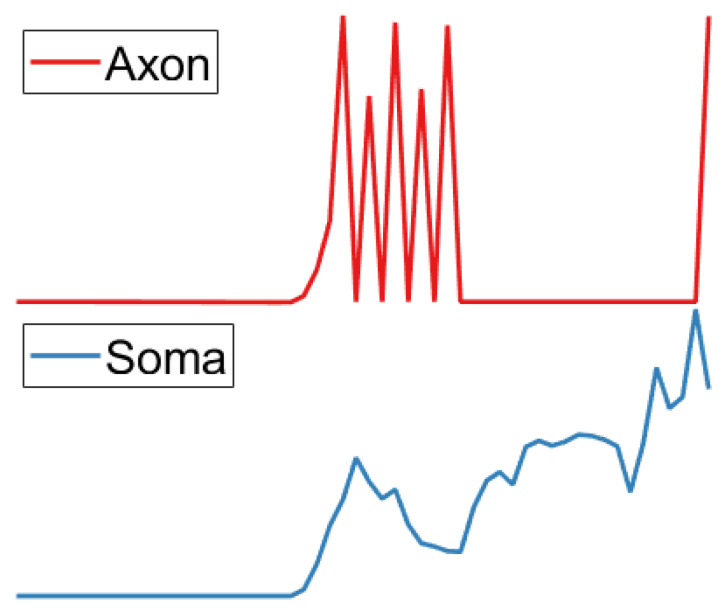
Soma layer input to the Izhikevich model produces an impulse sequence.

Based on the evidence that the generation of action potentials in LGMD neurons necessitates a prolonged period of high-potential pulse [20], an Izhikevich class 2 excitability model [27] has been devised to produce high-potential pulse signals. This model produces high-potential pulse signals, which we subsequently utilize as the present input to the Izhikevich neuron model in the Soma layer. The pulse sequence generated by the Axon layer is shown in Figure 6. The mathematical expression of the model is provided below:

(14) $$\left\{\begin{array}{c} \frac{dv}{dt}=0.04v^{2}\left(t-1\right)+5v\left(t-1\right)+140-u\left(t-1\right)+Soma\left(t-1\right)\\ \frac{du}{dt}=a\left(bv\left(t-1\right)-u\left(t-1\right)\right) \end{array}\right.$$

(15) $$v\left(t\right)=\left\{\begin{array}{cc} \frac{dv}{dt}\Delta t+v\left(t-1\right) & V<30mv\\ c & otherwise \end{array}\right.$$

(16) $$u\left(t\right)=\left\{\begin{array}{cc} \frac{du}{dt}\Delta t+u\left(t-1\right) & V<30mv\\ u(t-1)+d & otherwise \end{array}\right.$$

where *u* is the membrane recovery variable, comprising activation of the K-ion current and deactivation of the Na-ion current. *v* represents the membrane potential, and the expression of $0.04v^{2}\left(t-1\right)+5v\left(t-1\right)+140$ was selected based on the behavioral characteristics of a pulsed signal, which is a nonlinear activation, and the resulting magnitude to guarantee that the membrane potential is measured in mV and that time is measured in ms. An early warning signal is sent out when there is a spike in *v*. When the membrane potential *v* exceeds the threshold value of 30 mV, the model emits a pulse and resets the potential *v* as shown in Equation (15). Additionally, the model simulates the opening of a potassium channel during a decrease in action potential as demonstrated in Equation (16). Table 1 presents the conventional parameters.

## 4. Experiments and Results

Considering the complexity of real UAV flight environments, this paper focuses on the collision warning capability of A-LGMD in scenarios with a high probability of collision. This section presents a comprehensive evaluation of the A-LGMD model in various UAV flight scenarios both indoors and outdoors. The evaluation concentrates on the model’s capacity to extract visual cues, its sensitivity to background motion, and the warning time. Quantitative analysis was conducted to compare its performance with other models, demonstrating the efficiency of the proposed synaptic mapping and the robust real-time warning capability of the model.

### 4.1. Experimental Set-up

The stimulus input to the neural network comprised a simulated simulation and an actual first-person view (FPV) video of a drone in flight captured using OSMO Pocket (with a recorded video frame rate of 30 fps). The neural network was executed on a computer with a 2.1 GHz Intel Core i7 CPU and 16 GB of RAM.

### 4.2. Model Characteristic Analysis

For the performance of the A-LGMD model in synthetic looming scenarios, this experiment aimed to validate the capability of the A-LGMD model to extract the image velocity and acceleration in synthetic looming scenarios and its pre-collision warning performance. We constructed an OpenMV4 model using Matlab and the following camera parameters: a focal length of 2.8 mm, CMOS dimensions of 4.8 mm × 3.6 mm, a horizontal field of view of 70.8°, a vertical field of view of 55.6°, a frame rate of 30 fps, and image dimensions of 240 × 320 pixels.

**Figure 7:**
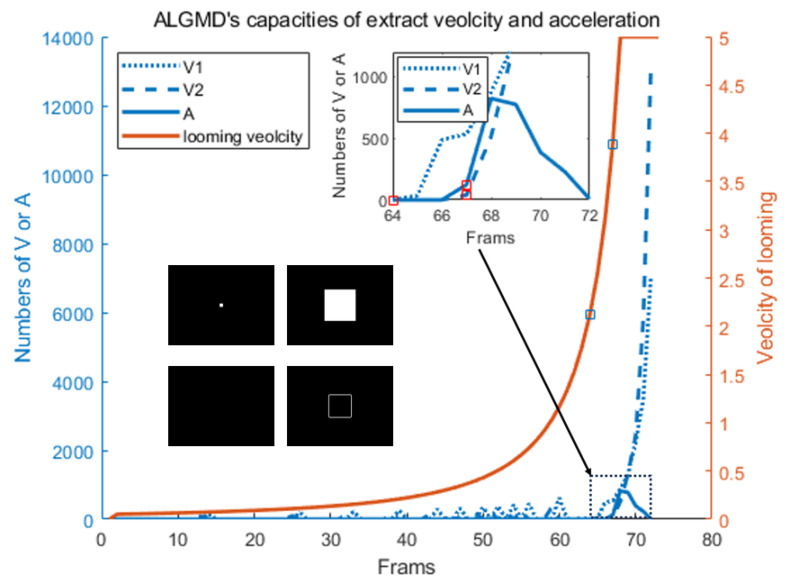
The capabilities of the A-LGMD model for extracting the angular speed and acceleration are demonstrated. The number of hazardous pixels in the velocity and acceleration layers is represented on the left y-axis, while the right y-axis displays the ideal magnitude of the angular velocity for a cube looming at a ratio of 0.1 ( l/v=0.1 ) per frame. During the 64th and 67th frames, the ideal angular velocities exceeded 60 and 120 pixels/s, respectively. Technical abbreviations are explained upon the first usage. The position changes of the cube in the field of view, and the variation in acceleration information extracted by the USTC layer of the model are illustrated in the diagram located in the lower left corner. The small diagram in the upper right corner offers a local magnification of the changing trend in the number of hazardous pixels in the velocity and acceleration layers of the A-LGMD neural network between frames 64 and 70.

In the experiment, a cube with a side length of 0.2 m was simulated. It was positioned at a distance of 5 m from the camera and moved toward it at a speed of 2 m per second until the cube’s edge reached the field of view’s edge as illustrated in Figure 7. According to the results of the experiment, the A-LGMD model is capable of effectively extracting the image motion velocity. Specifically, we extracted the values of velocities v1 and v2 from frames 65 and 68, respectively. In frame 68, a change in velocity was detected when the motion velocity of the image rapidly increased and exceeded v2’s threshold (120 pixels/s).

As the velocity of the cube surpassed v2 throughout the given timeframe, there was no area for acceleration. As a result, the model extracted angular acceleration data, displaying an abrupt peak within the angular velocity data from frames 67 to 70 and activating the alarm mechanism of the model’s Axon layer.

### 4.3. Real UAV Experiment

Finally, the model was tested on actual drone recordings, and indoor tests were carried out to assess the model’s performance in different obstacle textures and real collision scenarios. Several drone video sequences featuring collisions with various obstacles, including static chairs, QR code boards, off-road vehicles, and basketball poles, were collected, and the comprehensive information concerning the sequences can be found in Table 2. These experiments aim to evaluate the real-world applicability of the model.

#### 4.3.1. Indoor Simulated Collision Flight Experiments

**Figure 8:**
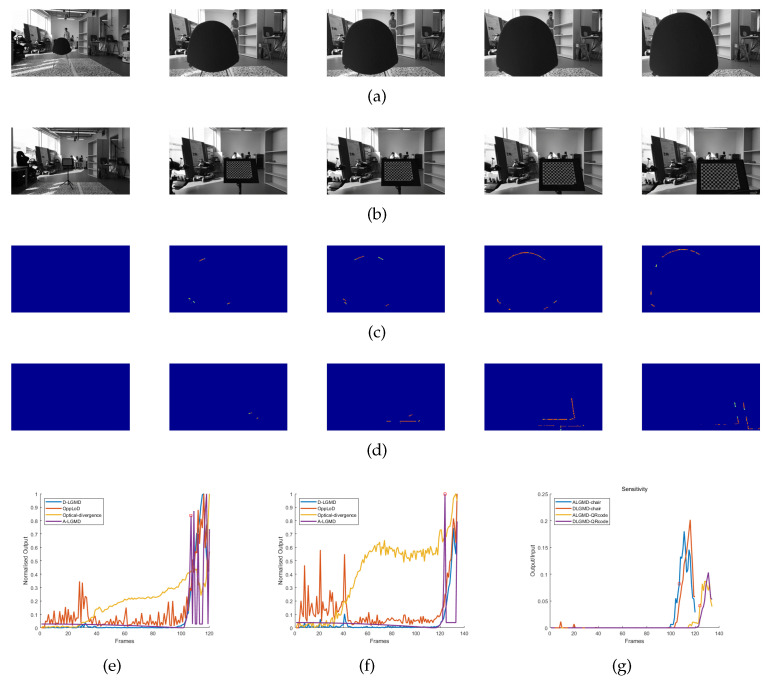
Indoor looming detection experimental data were recorded using cameras, and the performance of various looming detection models was evaluated. Grayscale images of Group 1 and Group 2 are shown in (**a**–**d**), displaying the output images of the A-LGMD model for Group 1 and Group 2 with corresponding sample images provided in Table 2. The normalized output curves of different models in the Group 1 and Group 2 datasets are presented in (**e**–**g**), showing the sensitivity of A-LGMD and D-LGMD to the looming region of the differential input image.

The datasets for Group 1 and Group 2 were obtained through the use of an OSMO Pocket camera while the drones were flown indoors. These experiments comprised five stages for the drones: hovering, pitching, accelerating, looming obstacles (looming), and program-controlled deceleration. Our experiments aimed to evaluate the performance of the A-LGMD algorithm on drones and the ability of biomimetic algorithms to adjust to varying obstacle textures. From Figure 8c,f, it is clear that the A-LGMD model efficiently extracted obstacle motion edges without being affected by the obstacle features. Furthermore, a sensitivity comparison test was conducted on the A-LGMD and D-LGMD models in response to the motion edges of a looming object during UAV motion. Sensitivity was defined using the following equation:

(17) $$Sensitivity\left(i\right)=Output_{looming}\left(i\right)/Input_{looming}\left(i\right)$$

where $Output_{looming}$ represents the number of pixel points depicted on the model’s resulting image within the looming region and $Input_{looming}$ corresponds to the number of pixel points depicted in the initial difference image within the same region. In Figure 8g, we can observe that the sensitivity of the A-LGMD model emerged earlier when compared with the D-LGMD model. Therefore, the alarm event of A-LGMD will occur before D-LGMD’s.

During the experiments conducted with Group 1 and Group 2, it was observed that both the OppLoD and Optical-divergence models registered a high number of false alarms. This occurrence can be attributed to the OppLoD model’s inability to accurately distinguish between the moving contours of the background and the symmetrical contours of the impending object during UAV motion, ultimately resulting in the generation of a false alarm signal. The high incidence of false alarms generated by the Optical-divergence model resulted from the strong influence of the background on the optical flow dispersion in the two selected regions. When the camera moved forward rapidly, the difference between the backgrounds of these regions caused a marked variation in the calculated optical divergence. As a result, this divergence was misinterpreted as a sign of an approaching object, leading to a false alarm situation.

#### 4.3.2. Outdoor Real Collision Tests

To assess the sensitivity of various biomimetic models in real collision scenarios, we used drone-captured videos as input data from a first-person perspective. Our objective was to evaluate their real-time performance by observing the warning and processing times and assessing their performance in identifying looming objects.

Groups 3, 4, and 5 depicted recordings of drones colliding in genuine flight circumstances. The A-LGMD model proved its capability to generate trustworthy early warning indicators (initiating avoidance commands while establishing the avoidance direction through the discerned motion contours of neighboring objects) during these experiments and portrayed the swiftest processing speed, rendering it more appropriate for the obstacle avoidance necessities of low-power drones operating in real time.

**Figure 9:**
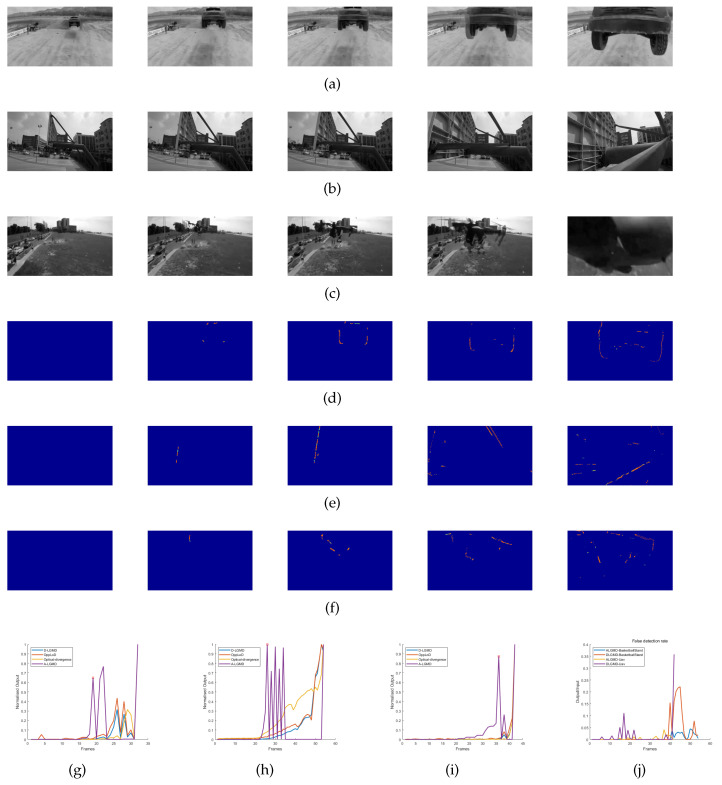
Experiments were conducted using real drone flights involving collisions, focusing on an objective perspective. Grayscale images from Groups 3, 4, and 5 are displayed in (**a**–**c**), respectively. In Group 3, a stationary drone collided with an off-road vehicle at high speed. In Group 4, the drone rapidly approached a basketball stand. In Group 5, a quadcopter collided with a uniformly moving drone. (**d**–**f**) The output images of the A-LGMD model in Groups 3, 4, and 5, respectively, with corresponding sample images listed in Table 2. (**g**–**i**) The normalized output curves of the A-LGMD model and other models in Group 3, 4, and 5 datasets. (**j**) The rate of false detections by A-LGMD and D-LGMD in the background section of the differential input image.

The normalized output curves indicate that both the D-LGMD model and the OppLoD model could perceive the oncoming UAV and basketball hoop. However, they exhibited early alarm times to collision (ATTCs) that were less than 0.18 s, as shown in Table 3. It is noteworthy that Group 3 barely recorded any near-collision incidents with the quadcopter. This implies that the D-LGMD and OppLoD models encountered challenges in delivering prompt cautionary notifications when genuine collisions were imminent. In addition, we conducted a comparison test between the A-LGMD and D-LGMD models for false detection backgrounds under the UAV’s motion, and we defined the false detection rate (FDR) as

(18) $$FDR\left(i\right)=Output_{background}\left(i\right)/Input_{background}\left(i\right)$$

where $Output_{background}$ represents the number of pixel points depicted in the model’s resulting image within the background region and $Input_{background}$ corresponds to the number of pixel points depicted in the initial difference image within the same region. Figure 9j shows that the FDR of the D-LGMD model was earlier and larger than that of the A-LGMD model. This implies that the persecution signals provided by the A-LGMD model were more trustworthy than those provided by the D-LGMD model. Based on this finding, combined with Figure 8g, we have reason to believe that the persecution motion information and warning information extracted by the A-LGMD model are reliable and valid. From the normalized output curves and the ATTC values in Table 3, it is evident that our proposed A-LGMD model was highly sensitive to forthcoming collisions. The model could detect the location of collision danger about 0.3 s before the collision occurred, consequently issuing a warning signal. Even in scenarios involving drone movement (Group 1, Group 2, Group 4, and Group 5), the A-LGMD model demonstrated efficient performance and displayed a significant advantage in ATTC when compared with other models, emphasizing its potential for widespread application in the drone industry.

### 4.4. Discussion

**Figure 10:**
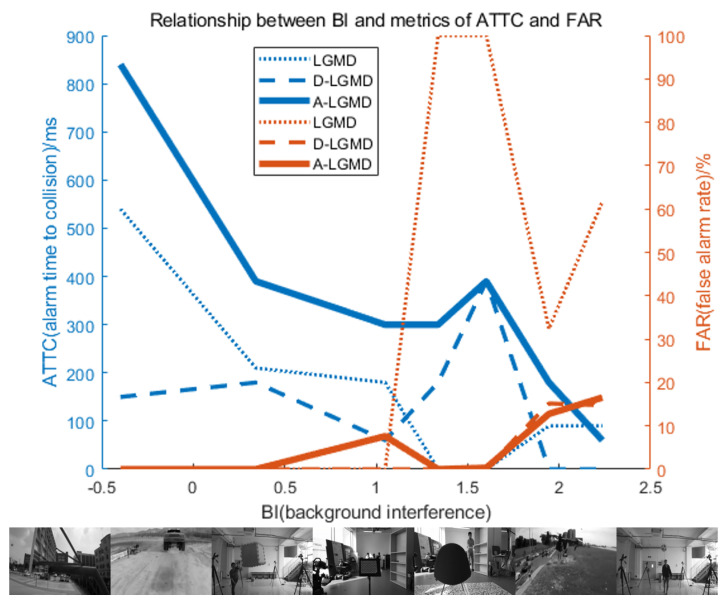
Alarm time to collision (ATTC) and false alarm rate (FAR) performance of different biomimetic looming detection models based on the field of view theory under different background interference (BI). The LGMD algorithm had a high false alarm rate when the UAV was moving. The D-LGMD algorithm was only sensitive to fast-moving objects and performed well in resolving the background interference caused by its motion, but this may result in a lower ATTC, which may not give the UAV enough time to avoid obstacles. In contrast, the A-LGMD algorithm had a high ATTC and a low false alarm rate, making it ideal for UAV looming detection.

This study examined the use of bio-inspired motion vision-based algorithms for detecting obstacles in autonomous drones and avoiding collisions, particularly in the presence of self-motion interference. The A-LGMD model was proposed based on an analysis of the characteristics of the angular acceleration information in images. The qualitative and quantitative analysis of the A-LGMD model, along with a comparison to other bionic approach detection models, validate the A-LGMD model’s capability to extract angular acceleration information and its feasibility for real-time early warnings, as shown in Figure 10, where the BI and FAR are defined as follows.

(19) $$BI=\frac{\int_{1}^{Frame_{end}}\frac{{Number}_{Background\;regeion\;in\;difference}}{{Number}_{Looming\;regeion\;in\;difference}}}{Frame_{end}}$$

(20) $$FAR=\frac{\int_{1}^{Frame_{alarm}}\frac{{Number}_{Background\;regeion\;in\;output}}{{Number}_{Output}}}{Frame_{alarm}}$$

Compared with other bio-inspired looming algorithms relying on motion vision, our algorithm incorporates higher-order motion information, specifically angular acceleration information, to alleviate interference caused by the observer’s self-motion and improve the perception of nearby objects. Furthermore, our caution system no longer relies on a singular threshold setting; instead, it incorporates numerous pieces of data and produces results via peaks in the output curve, resulting in heightened stability and dependability.

It should be noted that our current model cannot strictly extract an image’s angular acceleration. Instead, it only initially extracts the pixels of the image velocity jump as the “acceleration”. Improving the image angular velocity extraction may help obtain more accurate information on the image angular acceleration. Additionally, the model has many parameters that must be adjusted according to different scenarios. In the future, the model’s parameters could be combined with the regularity of natural scenes, and a deep learning method could be used to achieve adaptive adjustments based on different scenes.

## 5. Conclusions

In this paper, we present the A-LGMD neural network model, a bio-inspired proximity detection algorithm, to tackle the problem of UAVs experiencing delays in warnings or being unable to function during their motion. We introduced and examined the features of higher-order information on the angular size curves and integrated the angular acceleration information of images into proximity detection for the first time. This model can accurately extract information on image angular acceleration and fuse real-time multiple proximity visual cues to provide early warnings of looming objects of interest. The experiments conducted on the system indicate that the model can successfully extract the image angular acceleration, eliminate irrelevant background motion, forecast the ideal angular acceleration curve trend, and raise early alarms.

In conclusion, the prospects for the A-LGMD neural network model are promising. Several critical areas have been identified for further development and enhancement. Firstly, the scalability and computational efficiency of the A-LGMD model represent a key research focus. Advancements in methods to scale up the model, enabling it to handle larger datasets and computational workloads, will ensure efficient processing and real-time performance across diverse UAV environments. Integrating multi-sensor fusion techniques, such as cameras, lidar, and radar, into the A-LGMD model presents a significant opportunity to enhance its looming detection capabilities. This will improve the model’s adaptability to complex scenarios, making it more effective in real-world applications. Moreover, image angular acceleration data, a less easily perceivable high-order visual feature, could have a significant role in proximity detection and other areas of robot motion, including posture recognition and grasping tasks.

## Figures and Tables

**Table 1:** Constant parameters.

Parameter	Description	Value
r1	Slow inhibitory kernel’s radius in Equation (8)	1
r2	Fast inhibitory kernel’s radius in Equation (8)	2
k1	Activation function’s threshold in Equation (12)	0.03
k2	Activation function’s threshold in Equation (12)	0.1
k3	Activation function’s threshold in Equation (10)	0.1
a	Izhikevich model’s constants in Equation (14)	0.02
b	Izhikevich model’s constantsl in Equation (14)	0.2
c	Izhikevich model’s constants in Equation (15)	−65
d	Izhikevich model’s constants in Equation (16)	2

**Table 2:** Details of FPV image sequence.

Image Sequence	Background Complexity	Attitude Motion	Object Texture	Image Resolution	Collision Frame	Example of Frame Sequence
Compound Looming	None	None	White pixel	240*320	72	None
Group 1	Cluttered Surroundings	Pitch Accelerating	Pure Color Chair	240*320	120	[3,104,107,111,115]
Group 2	Cluttered Surroundings	Pitch Accelerating	Gridding Pattern	240*320	134	[3,120,124,129,134]
Group 3	Simple Surroundings	Static	Moving SUV	240*320	32	[3,17,19,24,26]
Group 4	Cluttered Surroundings	Low Speed Forward	Basketball Stands	240*320	54	[3,24,26,40,54]
Group 5	Cluttered Surroundings	High Speed Forward	Flying UAV	240*320	42	[3,32,36,40,42]

**Table 3:** Alarm time to collision (ATTC; unit = ms).

Image Sequence	D-LGMD	OppLoD	Optical Divergence	A-LGMD
Group 1	390	False	690	390
Group 2	180	False	False	300
Group 3	180	210	180	390
Group 4	150	150	570	840
Group 5	0	0	0	180

**Figure A1:**
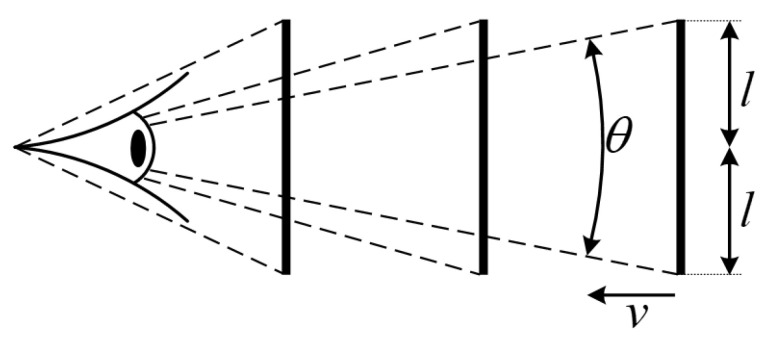
Object looming process. Black squares of different sizes approach at a constant speed and eventually collide with the observer. The object size and value close to the speed were considered to be constant, and thus $l_{v}$ = l/v was constant.

## Data Availability

The data of simulation and the source codes can be found via https://github.com/chasen-xqs/ALGMD (accessed on 6 October 2023).

## Appendix A. Problem Formulation

The visual system performs a fundamental function in biological perception and response. During an object’s approach, its area in the visual field progressively increases, a characteristic that can be simplified as the angle of the visual field expands. Gabbiani [7] derived a relationship between the process of change in the imaging angle and angular velocity of an obstacle in the retina and the ratio of the real size of the obstacle to the approximation velocity (refer to Equation (A1) and Figure 1). This relationship portrays the imaging angle and angular velocity change process of the obstacle in the retina, which provides an important theoretical basis for the encoding of the post-approaching visual cues. Based on this, Zhao [15] proposed the distributed presynaptic connection (DPC) structure to quantify the encoded image angular velocity and described the pre-collision process as a nonlinear increase in the image angular velocity. Additionally, collisions are reflected by the nonlinear increase in many image variation-based visual features that loom (such as dilatancy, radial symmetry, and angular velocity). However, the change in angular acceleration in higher-order visual fields is quite different, with a sharp peak in the angular acceleration curve and a linear correlation with the object size and motion speed before the TTC approaches zero, which is critical for early warnings of collisions. This section provides a detailed explanation of the need for angular acceleration to achieve early responses in collision detection algorithms:

(A1)
$$\theta \left(t\right)=\arctan\left(\frac{l_{v}}{t}\right)$$

To address image interference due to the robot’s movement and achieve a prompt response by the model, Equation (A1) denotes detecting obstacles when the object imaging angle theta is smaller so that the model can emit early warning signals for larger TTC values. Therefore, for the algorithm of detecting the angle, setting the threshold of theta to be smaller can theoretically achieve an early response, but this also leads the model to false alarms for various small objects, and the performance is not robust:

(A2)
$$\left\{\begin{array}{c} \dot{\theta }\left(t\right)=-\sin2\theta /2t\\ t=-\sin2\theta /2\dot{\theta }\left(t\right) \end{array}\right.$$

Based on the above analysis, following the derivation of Equation (A1), Equation (A2) provides the relationship between the TTC and image angular velocity and angle. The algorithm for detecting the angular velocity can achieve an early response by setting a small motion speed threshold when the object’s imaging angle is small, as demonstrated by Equation (A2). However, even slight winds in the scene or the robot’s motion caused by changes in the image can lead to false alarms and reduced model performance, thereby decreasing its robustness:

(A3)
$$\left\{\begin{array}{c} \ddot{\theta }\left(t\right)=-\sin2\theta \cos\left(2\theta +1\right)/2t^{2}\\ t=-\dot{\theta }\left(t\right)\cos\left(2\theta +1\right)/\ddot{\theta }\left(t\right) \end{array}\right.$$

By deriving Equation (A2), we can obtain the relationship between the image angular acceleration and angle as expressed in Equation (A3). The combination of the θ , $\dot{\theta }$ , and $\ddot{\theta }$ states can express the TTC, where the TTC is inversely proportional to $\ddot{\theta }$ and directly proportional to θ and $\dot{\theta }$ . If a model for detecting the angular acceleration exists, then a small angular acceleration threshold can be set during the detection of objects with small imaging angles θ and $\dot{\theta }$ . This would lead to an early response, indicating the importance of acceleration in estimating the TTC for looming detection:

(A4)
$$\left\{\begin{array}{c} \dot{\theta }\left(t\right)=-l_{v}/\left(l_{v}^{2}+t^{2}\right)\\ \ddot{\theta }\left(t\right)=2l_{v}t/{\left(l_{v}^{2}+t^{2}\right)}^{2}\\ \dddot{\theta }\left(t\right)=2l_{v}^{3}-6l_{v}t^{2}/{\left(l_{v}^{2}+t^{2}\right)}^{3}\\ \ddddot{\theta }\left(t\right)=24l_{v}t\left(t^{2}-l_{v}^{2}\right)/{\left(l_{v}^{2}+t^{2}\right)}^{4} \end{array}\right.$$

According to the higher-order field-of-view curves shown in Figure 1, the moment of the spike appears earlier with an increasing field-of-view curve order, and this is linearly related to $l_{v}$ . The expression for different higher-order field-of-view curves can be derived from Equation (A1) as shown in Equation (A4):

(A5)
$$\left\{\begin{array}{c} t_{spike2}=\frac{\sqrt{3}}{3}l_{v}\\ t_{spike3}=l_{v} \end{array}\right.$$

Let $\dddot{\theta }$ and $\ddddot{\theta }$ be zero. This provides us with the angular acceleration curves and the third-order field-of-view curve, with peaks occurring at times $t_{spike2}$ and $t_{spike3}$ as shown in Equation (A5). Consequently, if we can encode high field-of-view information, then we can provide collision warnings at the earliest possible moment, based on curves of spikes in the higher-order field-of-view.
